# Micrographic View of Graft Union Formation Between Watermelon Scion and Squash Rootstock

**DOI:** 10.3389/fpls.2022.878289

**Published:** 2022-04-15

**Authors:** Pinki Devi, Lisa DeVetter, Michael Kraft, Srijana Shrestha, Carol Miles

**Affiliations:** ^1^Department of Horticulture, Northwestern Washington Research and Extension Center, Washington State University, Mount Vernon, WA, United States; ^2^Scientific Technical Services, Western Washington University, Bellingham, WA, United States

**Keywords:** anatomy, cucurbit, hypocotyl, scanning electron microscopy, vascular connection

## Abstract

Grafting has become a common practice for watermelon [*Citrullus lanatus* (Thunb.) Matsum & Nakai] production in many parts of the world, due to its efficacy against biotic and abiotic stressors. However, grafting success for watermelon is challenging in part due to the complex anatomy of the cucurbit vascular system. The survival of grafted transplants depends on compatibility between the scion and rootstock, which in turn depends on anatomical, physiological, and genetic variables. A better understanding of cucurbit anatomy and graft union formation would inform grafting approaches and transplant management. An anatomical study was conducted by scanning electron microscopy (SEM) at 11 and 25 days after grafting (DAG) with seedless watermelon cultivar ‘Secretariat’ grafted onto compatible rootstock cultivars ‘Pelop’ (*Lagenaria siceraria*) and ‘Tetsukabuto’ (*Cucurbita maxima* × *Cucurbita moschata*) in comparison to non-grafted watermelon and rootstock seedlings. At 11 DAG, the parenchymatic cells of the central pith of grafted plants were dead and a necrotic layer was observed, representing the beginning of callus formation. New xylem strands were formed in the vascular system, connecting the rootstock with the scion. At 25 DAG, fully developed vascular bundles at the graft interface were observed with both scion-rootstock combinations. Although more studies are necessary to characterize the sequence of physiological events after grafting in Cucurbit species, this is one of the first studies to describe the complex anatomical changes that occur during watermelon graft healing.

## Introduction

Production of watermelon (*Citrullus lanatus*), an important Cucurbitaceae crop, has been adversely affected by soil-borne diseases such as fusarium wilt (caused by *Fusarium oxysporum* f. sp. *niveum*) and verticillium wilt (caused by *Verticillium dahliae*) (Bletsos et al., 2003; Bruton et al., 2007; Paroussi et al., 2007; Rivard and Louws, 2008). In recent years, grafting has become a common practice to protect watermelon against these diseases, especially in regions of the world where chemical fumigation is no longer permitted or is becoming increasingly restrictive (Traka-Mavrona et al., 2000; Yetisir and Sari, 2003; Cohen et al., 2007; Davis et al., 2008; Kubota et al., 2008; Fallik and Ziv, 2020). In addition to disease management, watermelon grafting has emerged as a promising biological management approach aimed at increasing tolerance to abiotic stresses such as salinity, saturated soil, and high or low temperatures (Yetisir et al., 2006; Cohen et al., 2007; Aloni et al., 2010; López-Marín et al., 2013; Kumar et al., 2017; Spalholz and Kubota, 2017).

Successful grafting is a complex structural and biochemical process whereby a continuous cambium and vascular system is created between the rootstock and scion (Olmstead et al., 2006). Failure to form a vascular connection leads to graft failure (Yeoman et al., 1978; Aloni et al., 2010; Melnyk, 2017). In grafted plants, scion-rootstock compatibility depends on anatomical, physiological, and genetic variables, which in turn impact the survival of grafted transplants. Within a graft union, the development and differentiation of vascular tissue varies with scion-rootstock combinations (Olmstead et al., 2006). In a successful graft union, three main steps are involved: (i) close contact of the cut tissues of the scion and the rootstock resulting in the formation of a necrotic layer with parenchymatous cells (callus) beginning to form within 1 or 2 days after grafting (DAG); (ii) cell differentiation takes place along with the formation of a continuous cambial connection between the rootstock and scion; and (iii) the vascular connection between the rootstock and scion is completed by the formation of a new cambial layer in the callus bridge tissue 7–10 DAG (Melnyk et al., 2015; Gautier et al., 2019; Frey et al., 2020; Rasool et al., 2020). The cells surrounding the graft interface also differentiate to vascular tissue along with the continuous cambial connection. Finally, the vascular connections are established between the rootstock and scion that remain throughout the life of the grafted plant (Martínez-Ballesta et al., 2010; Melnyk, 2017). Incompatibility is the failure of the scion to unite and adhere to the rootstock, rendering the plant unable to grow productively or contributing to premature plant death (Garner and Bradley, 2013; Melnyk, 2017). Although detailed molecular mechanisms of graft-union development are still unknown and require further research, some studies report that hormone classes such as auxin, cytokinin, and gibberellin play an integral role in regulating scion-rootstock interactions (Aloni et al., 2010).

Plant anatomy also has an integral role in grafting success. Although grafting is a simple process, it requires attention to the cut angle of both scion and rootstock so that vascular cambium tissues are properly aligned. Learning more about plant anatomy and early healing following grafting could inform grafting procedures and aid in the development of successful management for graft healing, particularly for cucurbit crops such as watermelon, melon (*Cucumis melo*) and cucumber (*Cucumis sativus*) that have a lower probability of grafting success than solanaceous crops such as tomato (*Solanum lycopersicum*), eggplant (*Solanum melongena*), and pepper (*Capsicum annuum*) (Oda, 2007; Johnson and Miles, 2011). Cucurbits have a complex vasculature that influences grafting success. Sieve elements are present in vascular bundles on both sides of the xylem (internal and external fascicular phloem) and there is also an extensive system of sieve tubes outside of the vascular bundles (extrafascicular phloem) (Crafts, 1932; Zhang et al., 2012). Severing the hypocotyl results in massive disruption of sieve element contents from the phloem and causes surging of the displaced phloem sap onto the sieve plate. Zhang et al. (2010) showed that in cucurbits, the fascicular phloem quickly becomes sealed when the hypocotyl is cut. As a result, sieve plate pores become plugged, making it difficult to stain the sample to observe under a microscope. The extrafascicular phloem forms an anastomosing (vein-like) network that interconnects the vascular bundles laterally in the stem and petiole. The extrafascicular phloem exudes profusely from cut stems and petioles, indicating that it does not have the normal sealing systems found in other types of phloem tissue (Van Bel and Gaupels, 2004; Turgeon and Oparka, 2010; Zhang et al., 2010, 2012). Furthermore, in cucurbits, deposition of callose to seal the sieve plate pores is slow in response to wounding (Mullendore et al., 2010; Zhang et al., 2012).

Learning more about plant anatomy during graft union formation could inform grafting approaches and grafted seedling management to increase the success of cucurbit grafting. For example, Aloni et al. (2008) tested compatible (‘RS59’) and incompatible (‘RS62’) Cucurbita hybrid cultivar rootstocks with melon cultivar ‘Arava’ scion to identify physiological and biochemical factors in the scion-rootstock interface that could be associated with graft compatibility. At 14 DAG there were no differences in water uptake and sugar distribution between the plant canopy and the roots for the compatible and incompatible combinations. At 24 DAG, however, both water uptake and root-sugar concentrations decreased significantly in the incompatible rootstock and the stem of the rootstock collapsed. A physical barrier might form between incompatible plants within 2 weeks after grafting, which may be responsible for cellular degradation in the grafting zone and rootstock failure. The vascular connection between the rootstock and scion influences water and nutrient translocation, affecting other physiological traits such as lateral and vertical development of roots, uptake ability of water and nutrients, stomatal conductance, and scion growth (Atkinson and Else, 2001; Oda et al., 2005; Martínez-Ballesta et al., 2010).

Previous research suggests morphological and anatomical changes at the graft-union interface can provide information about the compatibility or incompatibility of rootstock-scion combinations. Given this, the aim of this study was to elucidate the early processes that occur at the interface between watermelon scion grafted with compatible rootstocks using scanning electron microscopy (SEM), and compare to non-grafted watermelon and rootstock seedlings. SEM micrographs show the three-dimensional morphology of anatomical structures and are used in this study to characterize the early developmental stages following grafting (Franks, 2014).

## Materials and Methods

### Experiment Location

This study was carried out in 2019. Watermelon seedlings were grafted at the Washington State University Northwestern Washington Research and Extension Center (WSU NWREC) greenhouse facilities in Mount Vernon, Washington. SEM micrograph analysis was carried out at the Western Washington University (WWU) SEM laboratory in Bellingham, WA, which is located about 45 km north of WSU NWREC.

### Plant Material

Seedless watermelon cultivar ‘Secretariat’ (Sakata Seeds America, Inc., Morgan Hill, CA, United States) was used as the scion and was grafted with two compatible rootstock cultivars ‘Pelop’ (*Lagenaria siceraria*; Rijk Zwaan, Salinas, CA, United States) and ‘Tetsukabuto’ (*Cucurbita maxima* × *Cucurbita moschata*; American Takii, Salinas, CA, United States). Scion and rootstocks were sown into 72-cell trays filled with potting mix (Sunshine #3 N&O; Sun Gro Horticulture, Agawam, MA, United States). For the grafted treatments, ‘Secretariat’ was seeded on 17 July and ‘Pelop’ and ‘Tetsukabuto’ were seeded in every other row of the seedling trays (to optimize the healing microclimate) on 23 and 26 July, respectively. The three seeding dates were staggered so that all of the cultivars reached the appropriate size for grafting (i.e., fully developed first true-leaf stage for scion material and early first true-leaf stage for rootstock material) at approximately the same time (Miles et al., 2017). Non-grafted treatments were seeded later so that non-grafted seedlings would be similar in size as the grafted plants on the date of SEM. ‘Secretariat’ was seeded on 1 August and ‘Pelop’ and ‘Tetsukabuto’ were seeded on 9 August, and SEM occurred 15 days after seeding for non-grafted ‘Secretariat’ and 7 days after seeding for non-grafted ‘Pelop’ and ‘Tetsukabuto’. There were 30 plants seeded for ‘Secretariat’, 20 plants seeded for each rootstock, and 12 plants were grafted for each of the rootstocks. For each treatment, three plants were analyzed at each sampling time.

### Grafting Methods and Healing

The one-cotyledon grafting method was used for this study and plants were grafted on 4 August, from 7:30 a.m. to 11:00 a.m. to minimize water stress (Miles et al., 2016). The rootstock seedlings were cut at about a 60° angle such that one cotyledon remained and the other cotyledon and its growth point were removed. The scion seedlings were cut at a 60° angle approximately 2 cm below the cotyledons such that hypocotyl diameters of rootstocks and scions were similarly sized. The two cut hypocotyl surfaces were then placed together and held with a grafting clip (3 mm; Johnny’s Selected Seeds, Fairfield, ME, United States).

Immediately after grafting, the plants were placed in a healing chamber on a bench in the greenhouse and followed a 9-day site-specific protocol (Miles et al., 2016), which is summarized here. The healing chamber was covered with one layer of clear plastic (0.15 mm polythene; Ginegar Plastic Products, Ginegar, Israel), and a thin film of water was added to the floor of the chamber to attain 100% relative humidity (RH) inside the chamber when it was closed. The chamber was covered with a secondary layer of black fabric (Contractor Landscape Fabric; American Nettings & Fabric, Ferndale, WA, United States) to limit light penetration to the plants. Daily average temperature inside the healing chamber ranged between 23 and 28°C and the daily average temperatures in the greenhouse ranged between 21 and 27°C. Daily average RH during this time was 94–98% inside the chamber and 50–72% in the greenhouse. Plants were sealed in the chamber to maintain complete darkness for 1 and 2 DAG. On 3 DAG, the chamber was opened for 5 min and water was added to the chamber. On 4 DAG, the chamber was opened for 15 min and the light level was increased by folding up the black fabric to expose the front side of the chamber. On 5 DAG, the chamber was opened for 30–45 min and water was added to the floor of the chamber and the black fabric was folded up halfway from all the sides of the chamber. On 6 DAG, the chamber was kept open for 1.5 h and only the top of the chamber was covered with black fabric. On 7 and 8 DAG, the chamber was opened for 4 and 6 h, respectively, and water was added to the floor of the chamber, and grafted plants were misted with water. Plants were removed from the chamber on 9 DAG and placed on greenhouse benches and watered following common greenhouse practice.

### Anatomical Study

**FIGURE 1:**
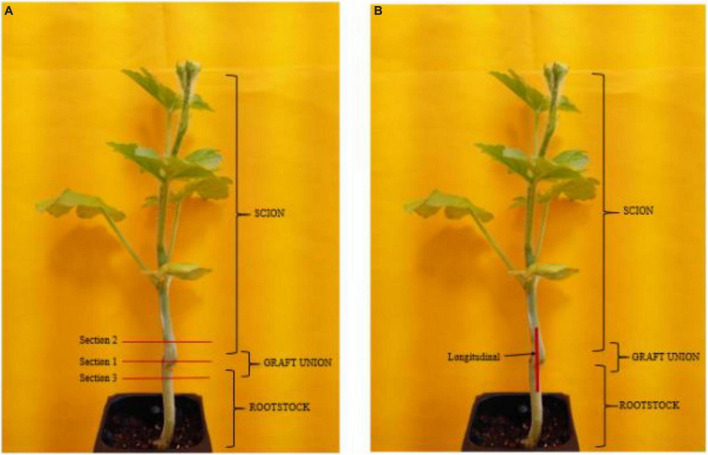
Transverse **(A)** and perpendicular **(B)** hypocotyl samples of watermelon cultivar ‘Secretariat’ grafted on rootstock cultivar ‘Tetsukabuto’.

**FIGURE 2:**
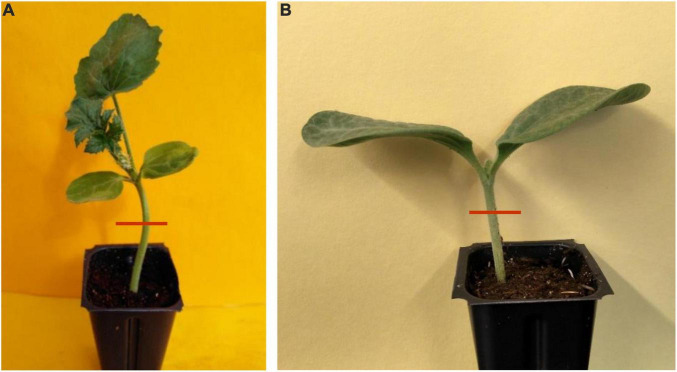
Cross-section hypocotyl samples of non-grafted watermelon cultivar ‘Secretariat’ **(A)** and rootstock cultivar ‘Tetsukabuto’ **(B)**.

On 15 and 29 August, 11 and 25 DAG respectively, plants were transferred to WWU where samples were prepared in the SEM laboratory. The sampling times were chosen to represent the early and complete formation of the graft union between the rootstock and scion. Cut samples were collected for the two grafting combinations, ‘Secretariat’ grafted on ‘Tetsukabuto’ and on ‘Pelop’, and from non-grafted watermelon and rootstock seedlings. For the grafted plants, at both sampling times, samples were collected from two sets of plants: the hypocotyls of one set of plants were cut in three transverse sections through the graft union, and the hypocotyls of the second set of plants were cut perpendicular to the cotyledons in the center of the hypocotyl (Figure 1). The three cross-sections were: (1) top of the graft union, (2) centered through the graft union, and (3) bottom of the graft union. For non-grafted watermelon and rootstock plants, a transverse section was cut at approximately the same region of the hypocotyl where grafted plants were sampled (Figure 2). Three replicates for each sample at each sampling time were prepared. Samples were cut with a clean, sharp razor blade and placed on a pin mount specimen holder using adhesive Pelco^®^ Colloidal Graphite (isopropanol base) (Ted Pella, Inc., Redding, CA, United States). For imaging, no fixation nor coating was required for the sample based on a preliminary test. Viewing and imaging were performed using a Tescan Vega 3 SEM (S-570 LaB6; Hitachi, Tokyo, Japan) in variable pressure mode (25 Pa of N_2_ gas in the specimen chamber and beam energy of 20 keV). Images were collected using the back-scattered electron (BSE) detector and low-vacuum secondary Tescan detector (LVSTD). Composite BSE-SE images showed features better than either BSE or SE alone. Image resolution was high enough to allow the visualization of the graft union interface of the samples.

## Results and Discussion

**FIGURE 3:**
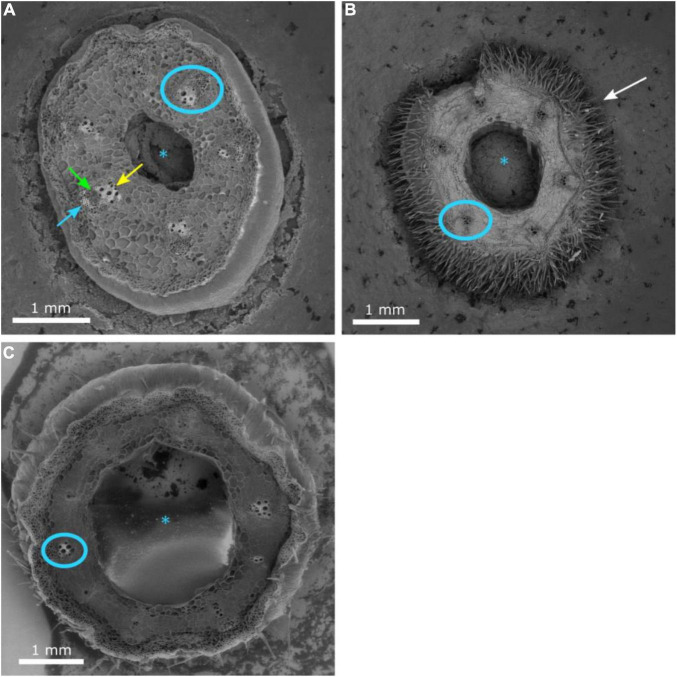
Cross-section of the hypocotyl of non-grafted watermelon cultivar ‘Secretariat’ **(A)** at 15 days after seeding and rootstocks ‘Pelop’ **(B)** and ‘Tetsukabuto’ **(C)** 7 days after seeding. The pith (asterisk), mature vascular bundle (blue circle), xylem (green arrow), internal phloem (yellow arrow), external phloem (blue arrow), and epidermal hairs (white arrow) are indicated.

**FIGURE 4:**
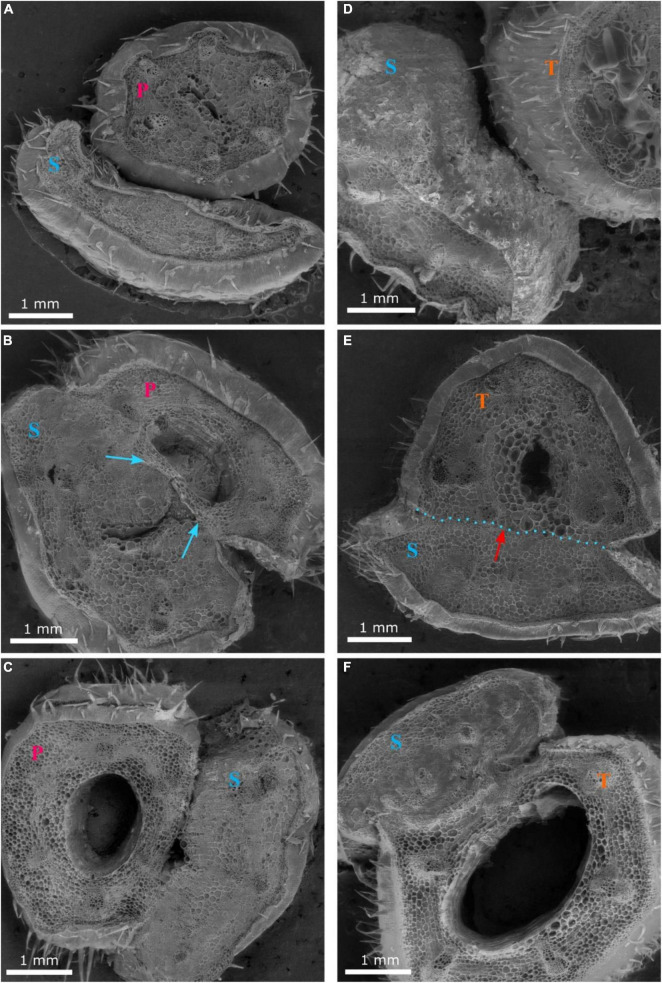
Cross-sections 1 **(A,D)**, 2 **(B,E)** and 3 **(C,F)** of the graft union between watermelon cultivar ‘Secretariat’ (S) and rootstocks ‘Pelop’ (P; **A–C**) and ‘Tetsukabuto’ (T; **D–F**) at 11 days after grafting; where cross-sections were: (1) top of the graft union, (2) centered through the graft union, and (3) bottom of the graft union. The blue arrow **(B)** indicates dead parenchymatic cells at the pith center and a necrotic layer. The red arrow with blue dotted line **(E)** indicates new xylem strands are formed in the vascular system and connect the rootstock with the scion.

**FIGURE 5:**
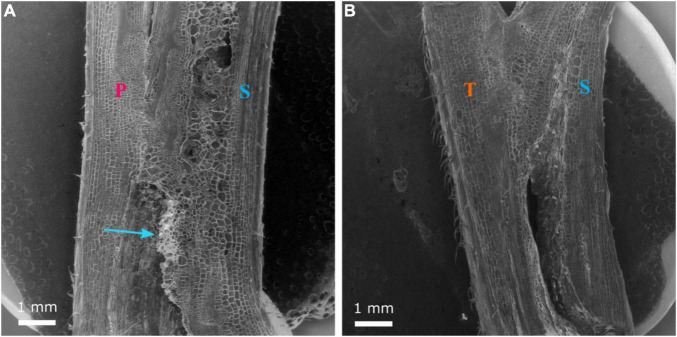
Longitudinal section of the graft union between watermelon cultivar ‘Secretariat’ (S) grafted onto rootstock ‘Pelop’ (P; **A**) and ‘Tetsukabuto’ (T; **B**) at 11 days after grafting. Parenchymatous callus has formed at the graft interface and the blue arrow **(A)** indicates a non-uniform layer of sugar substances.

Grafting watermelon is an effective strategy for managing biotic and abiotic stress, and account for 95–100% of cultivated watermelon production in some countries (Devi et al., 2020). Increased grafting success can lead to decreased cost for grafted transplants, which will lead to increased use in regions where the economic returns from grafting are questionable (Djidonou et al., 2013; Galinato et al., 2016). Earlier studies in vegetable grafting show that the anatomical changes that occur during the formation of a graft union include the death of cell layers at the graft interface, cohesion of the scion and rootstock through callus formation from parenchymatous cells of xylem and phloem tissue, proliferation of callus cells at the graft interface, and vascular differentiation across the graft interface to establish vascular continuity (Hartmann et al., 2002; Martínez-Ballesta et al., 2010). In the current study, the hypocotyl cross-section of non-grafted ‘Secretariat’ at 15 days after seeding, and rootstocks ‘Pelop’ and ‘Tetsukabuto’ at 7 days after seeding are presented in Figure 3 while Figure 4 shows the three hypocotyl cross-sections of ‘Secretariat’ grafted onto ‘Pelop’ and ‘Tetsukabuto’ at 11 DAG. Detailed structural views of the vascular bundle, cambium, pith, and epidermal hairs were observed for all samples. At 11 DAG, several developmental stages can be recognized in the formation of the graft union between the watermelon scion and the squash and bottle gourd rootstocks. The parenchymatic cells of the central pith were dead and a necrotic layer was observed (Figure 4B), which represents the beginning of the undifferentiated cell callus development in response to wounding. At this stage, new cambial differentiation begins, with callus proliferation at the graft interface. Further, new xylem strands were formed in the vascular system and connect the rootstock with the scion (Figure 4E). At 11 DAG, the newly formed parenchymatous callus at the graft interface were covered by a non-uniform layer of sugar substance including pectin (Figure 5). This might indicate the callus proliferation at the graft interface, with non-ordered histological rearrangement of scion-rootstock cells. After the graft assemblage between the cells of the rootstock and scion is formed, differentiation of the new vascular system begins.

**FIGURE 6:**
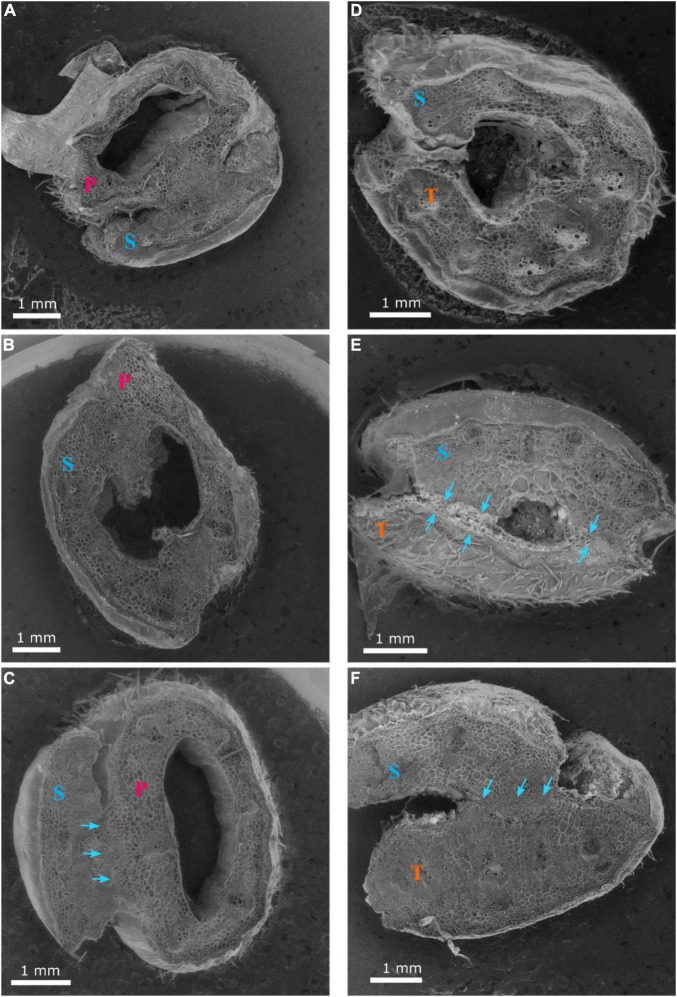
Cross-sections 1 **(A,D)**, 2 **(B,E)** and 3 **(C,F)** of the graft union between watermelon cultivar ‘Secretariat’ (S) and rootstocks ‘Pelop’ (P; **A–C**) and ‘Tetsukabuto’ (T; **D–F**) at 25 days after grafting; where cross-sections were: (1) top of the graft union, (2) centered through the graft union, and (3) bottom of the graft union. The blue arrows **(C,E,F)** indicate the fully developed xylem vessels connecting the rootstock and scion at the graft union.

**FIGURE 7:**
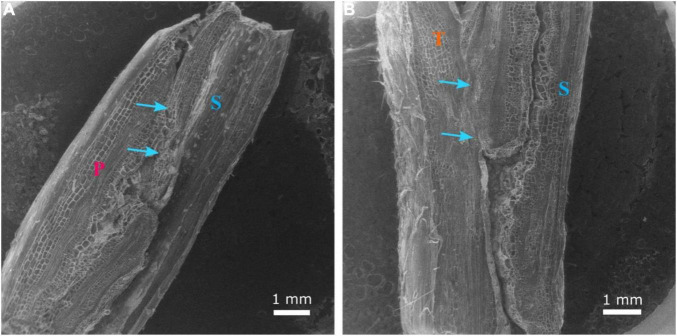
Longitudinal section of the graft union between watermelon cultivar ‘Secretariat’ (S) grafted onto rootstock ‘Pelop’ (P; **A**) and ‘Tetsukabuto’ (T; **B**) at 25 days after grafting. The scion/rootstock tissue union was complete, with xylem vessels connecting rootstock and scion (blue arrows).

The three hypocotyl cross-sections of ‘Secretariat’ grafted onto ‘Pelop’ and ‘Tetsukabuto’ at 25 DAG are shown in Figure 6. At 25 DAG, fully developed xylem vessels connected the rootstock and scion at the graft union (Figures 6C,E,F). In grafted watermelon plants, the callus was formed by all living, undamaged cells at the graft union, probably from cambial, ray parenchyma, and phloem parenchyma cells. Previous studies have considered a graft union to be successful and complete when several phloem and xylem connections cross the graft interface through cytoplasmic cell connections (plasmodesmata) whose primary or secondary formation has been documented in various plant systems (Botha and van Bel, 1992; Roberts and Oparka, 2003; Fernández-García et al., 2004; Aloni et al., 2008; Martínez-Ballesta et al., 2010). Similar observations are shown in Figures 6,7. Clear scion/rootstock tissue differentiation were observed at 25 DAG with the graft union complete at the grafting interface (Figure 7). This leads to vascular rearrangement and new cambium establishment, which can provide the water and nutrient flow and cell-to-cell communication between the grafted watermelon scion and rootstock, ensuring the success of the grafting process.

This study supports the general finding that graft healing has commenced in watermelon by 11 DAG, as demonstrated by the general practice of removing grafted watermelon transplants from the healing chamber at 10 DAG (Davis et al., 2008). Our study showed that callus proliferation had taken place with non-ordered histological rearrangement between the scion and rootstock by 11 DAG. Additionally, there are fully developed vascular bundles at the graft interface by 25 DAG. Although adhesion of vascular bundles and subsequent callus proliferation during the first two weeks following grafting in *Prunus* spp. may appear between compatible scion/rootstock combinations, these steps could also occur between incompatible grafting combinations but not be evident until later (Pina et al., 2012). Other studies indicate new cambium derived from the callus tissue is formed across the graft interface, and several phloem and xylem connections that cross the graft interface are crucial for the successful and complete graft union (Fernández-García et al., 2004; Trinchera et al., 2013). Further, compatible grafting combinations with watermelon scion appear to have a longer adhesion region (the area of callus formation between the scion and rootstock) than incompatible combinations at 15 DAG (Kaseb et al., 2021). In another study, morphological changes occurred earlier on the scion side than on the rootstock side in graft unions between cucumber (*C. sativus* ‘Zhongnong NO.26’) on pumpkin rootstock (*C. moschata* ‘Jingyan NO.5’) using the hole-insertion, tongue-insertion, or splice-grafting with one-cotyledon methods. Further, plants grafted with the hole- and tongue-insertion methods had vascular bridge formation 2 days earlier than splice-grafted plants (Miao et al., 2019).

The formation of new cambium at the graft union appears to be earlier for other crops. For example, in tomato, the formation of xylem and phloem vessels through the graft union was evident in just 8 DAG (Fernández-García et al., 2004). More research is needed to compare the morphological changes in plants grafted with compatible and incompatible rootstocks. It is interesting to note that watermelon has seven vascular bundles while the most popular rootstocks for grafting watermelon have six (*C. maxima* × *C. moschata*) or eight (*L. siceraria*) vascular bundles, indicating that successful vascular alignment occurs even though number of vascular bundles differ between scion and rootstock (Yetisir and Sari, 2004). To better understand morphological changes that occur during graft healing, observations are needed from 3 DAG to 30 DAG, which is during the early processes of healing and until the vascular bundle system is fully developed at the graft interface. Future research should also compare the graft healing process between solanaceous and cucurbit plants during each graft healing stage to advance knowledge of graft healing duration differences anatomically and morphologically within both these crops. Studies should also compare a diversity of scion-rootstock combinations grown with different climatic conditions to assess if there is an impact on the timing of the stages of graft union formation. These studies would provide a better understanding of the sequence of physiological pathways during graft healing and if the mechanisms of graft healing can be influenced by climate factors.

## Data Availability Statement

The raw data supporting the conclusions of this article will be made available by the authors, without undue reservation.

## Author Contributions

PD and CM conceived and designed the experiments and wrote the original draft. PD and MK conducted the laboratory analyses. All authors provided the editorial advice, and reviewed and revised the manuscript.

## Conflict of Interest

The authors declare that the research was conducted in the absence of any commercial or financial relationships that could be construed as a potential conflict of interest.

## Publisher’s Note

All claims expressed in this article are solely those of the authors and do not necessarily represent those of their affiliated organizations, or those of the publisher, the editors and the reviewers. Any product that may be evaluated in this article, or claim that may be made by its manufacturer, is not guaranteed or endorsed by the publisher.

## Abbreviations

DAG: days after grafting.
